# A Cohort Study of the Diversity in Animated Films From 1937 to 2021: In a World Less Enchanted Can We Be More Encanto?

**DOI:** 10.7759/cureus.43548

**Published:** 2023-08-15

**Authors:** Suneil A Raju, Samira R Sanders, Kathryn S Bolton-Raju, Freya J Bowker-Howell, Lara R Hall, Millie Newton, Gary S Neill, William J Holland, Katie L Howford, Emma V Bolton, Pranav Arora, Aneil S Raju, Premal J Shah, Iman Ahmed F Azmy, David S Sanders

**Affiliations:** 1 Department of Infection, Immunity and Cardiovascular Diseases, The University of Sheffield, Sheffield, GBR; 2 Academic Unit of Gastroenterology, Sheffield Teaching Hospitals National Health Service (NHS) Foundation Trust, Sheffield, GBR; 3 Department of History, The University of Sheffield, Sheffield, GBR; 4 Division of Psychiatry, The University of Edinburgh, Edinburgh, GBR; 5 Department of Breast Surgery, Chesterfield Royal Hospital National Health Service (NHS) Foundation Trust, Chesterfield, GBR

**Keywords:** culture and media, physical literacy, mental health literacy, overweight and obesity, racial and ethnic disparities, women in leadership, diversity and inclusion

## Abstract

Background

Exposure to gender stereotypes in the media can develop and reinforce these attitudes in children. Individuals who are overweight, have health conditions, or are from a minority ethnic group (IMEG) are both underrepresented and poorly portrayed in the media. Role models can raise the aspirations of young children both professionally and in taking ownership of their health. We aimed to assess how the portrayal and diversity of characters in Disney, Pixar, and Dreamworks animated films have changed over time.

Method

A cohort study of all main characters in Disney, Pixar, and Dreamworks feature-length, theatrical, animated films from 1937 to 2021 was conducted. The portrayal of characters (R-score divided into negative, neutral, and positive -1, 0, and 1, respectively) was scored. The proportion of individuals with certain protected characteristics (sex, increased body mass index, physical or mental health conditions, being from an IMEG or part of the lesbian, gay, bisexual, transexual, and queer community) was also recorded.

Results

In total, 116 films and 1,275 characters were included. From the 1930s to 2020s, the proportion of women in films increased (16.7% to 47.3%, p=0.008) and their representation was more positive (mean R-score = -0.10 (SD:0.692) versus 0.49 (SD:0.837), p<0.001, respectively). The portrayal of overweight individuals has improved to a neutral position (mean R-score: -0.67 to 0.0). Both physical and mental illnesses are better portrayed (mean R-score: -0.18 to 0.34, p=0.004 and 0.5 to 1.0, p= 0.019, respectively). IMEGs introduced in 1953 now play more than just negative roles (mean R-score = -1 to 0.76, p=0.008).

There is only one explicitly stated homosexual character. The most diverse film is Encanto.

Conclusion

This is the first study to comprehensively assess the diversity of animated film characters. We have identified an improvement in diversity and the way diverse individuals are portrayed which we hope continues.

## Introduction

From the late 1920s onwards, Walt Disney created a wealth of animated films and founded a company now one of the largest in the world [1]. Its animated films are seen by millions of adults and children. However, despite their significant reach, the messages portrayed in these animated films about stereotypes, diversity, and ableism have not been explored.

Exposure to gender stereotypes in the media can develop and reinforce such attitudes in children; therefore, the messages conveyed in films targeting children must be carefully considered [2]. Rutherford and Baker identified the “princess bubble” as Disney Princesses who were flawlessly beautiful women with an “ideal” figure that is thinner than the perceived current body size [3,4]. These princesses are also subject to what Xu et al. described as the “Cinderella complex,” in which they are dependent on men to overcome their challenges [5]. A small study of only Disney Princesses (n=13) found that their depiction of role models is gender-stereotyped and can potentially affect the self-esteem of children [3,6]. When considering real-life disparities in diversity, women in the NHS workforce remain less represented than men in senior positions, and the same applies in the areas of science, technology, engineering, and mathematics (STEM) [7,8].

As “weight diversity” becomes a greater social and political issue, campaigners say they have a human right to be overweight [9-11]. In the United Kingdom, the median weight of a 10-year-old child is on the 67th percentile of the 1990 baseline distribution, yet over two-thirds of parents describe their overweight child as of “normal weight” [12,13]. This increased body mass index, which has been normalized, is associated with many health conditions, reduced productivity, and higher healthcare costs [14,15]. There are multiple factors attributed to the growing obesity epidemic including increased availability of high-fat and added-sugar foods, social conditioning, media coverage of obesity, reduced physical activity, and advertising of unhealthy foods [16-19]. Despite the recognition of the media's influence on body image, to our knowledge, this has not been explored in films aimed largely at children.

Individuals with disabilities are underrepresented in media. Furthermore, there is an under-representation of disabilities among academic staff. In 2012/2013, the prevalence of university staff declaring health conditions was 3.9% [20]. However, 16% of working-age adults and nearly 13% of undergraduates have a known disability [20].

Within medical education, there exists a perceived negative stereotype of individuals from a minority ethnic group (IMEG) which jeopardizes the relationship with educators and subsequent exam performance [21,22]. This disadvantage can also be seen in medical school’s admissions processes [23]. Historically, the media has played a role in creating a negative image of IMEG; however, their representation in feature-length animated films has not previously been explored [24,25].

Health disparities have significant social and economic costs to both individuals and societies [26]. In the United Kingdom, significant disparities in life expectancy can be seen over short distances; for instance, in London, each eastbound tube station passed from Westminster represents nearly one year of life expectancy lost [26]. A diverse workforce helps care for an increasingly diverse population and, therefore, needs to be encouraged [27]. While diversity in the workplace has increased over time, the majority of people of color in healthcare jobs remain in entry-level and lower-paying jobs [28]. Furthermore, in the United Kingdom, despite 61% of pharmacists being female, only 36% hold senior positions [29]. While this problem is likely multi-factorial, we aimed to assess the possible impact role models may have on this.

For these reasons, we aimed to assess diversity over time in feature-length, animated films by Disney, Pixar, and Dreamworks. The null hypothesis was that there was no change in diversity or the way diverse characters are portrayed over time.

## Materials and methods

The cohort included all Disney, Pixar, and Dreamworks studios' feature-length, theatrical, animated films. Feature length was defined as films longer than 40 minutes, as per the Academy of Motion Picture Arts and Sciences [30]. Films were identified using the peer-reviewed online resource Wikipedia [31-33]. Feature-length films with US theatrical release dates within the study time period, January 1937 to November 2021, were included. Live-action and package films were excluded. Eight items were agreed upon to be assessed based on the Equality Act 2010 protected characteristics: species, gender, obesity, physical health, mental health, ethnicity, sexuality, and whether they were a role model [34]. Characters were included in the assessment if deemed significant, defined as appearing in more than five scenes with dialogue or having a significant effect on the storyline (analogous to the Bechdel test) [35-37].

Each character was then scored a maximum of 1 per category if they were not male, had a physical health condition/disability or had a mental health condition, were not Caucasian, and were not heterosexual, producing a character score (C-score: maximum score possible is 5). A physical health condition was defined as an individual having a long-standing illness, disability, or impairment causing difficulty with day-to-day activities as per the Equality Act 2010 [34]. Patients considered to have a mental health condition were identified by reviewers. They were then independently reviewed by a qualified senior psychiatrist with over 25 years of practical experience in mood disorders, psychotic disorders, anxiety disorders, and personality-related problems and membership with the Royal College of Psychiatrists. The Diagnostic and Statistical Manual of Mental Disorders, Fifth Edition (DSM-V) was used as a reference [38].

Observations were made by the authors. To score a point, each characteristic measured must have been observable or explicitly stated beyond a reasonable doubt. Where there was doubt, the point was not given, as if a group of interested reviewers could not identify the characteristic, it was felt unlikely the general viewer when casually viewing would. A character’s sexuality was assumed heterosexual unless explicitly stated otherwise, in this way undisputable diverse representation could be measured as opposed to implied diversity.

The role model category was scored as negative, neutral, or positive (R score = -1, 0, or +1, respectively). If characters were identified as being overweight, we then assessed their R-scores.

By multiplying the C-score by the R-score, we created an individual fairy tale representation (FTR) score. The FTR scores for each character in a film were then totaled to compare films against each other. A further score that doubled the weighting of main characters was also used (FTRC) score. This gave a ranking for diversity across all the films included.

Six groups of two investigators were randomly assigned an even number of films per decade (by a random number generator). Five percent of films were the same in all six investigator teams in order to measure agreeability amongst investigators (the investigators were blinded to this). Assigned films were reviewed by each group, and data was recorded in a data collection tool using Microsoft Excel (Microsoft 365, version 2304, Washington, USA). Consensus meetings occurred prior to study commencement after 10% of films had been reviewed (in order to discuss any challenging characters and assure reviewers were assessing in the same way) and finally upon completion of all films to assure reliability within the study. Any disagreements were resolved through discussion between all reviewers. Fleiss’ kappa was run to determine the inter-rater agreement between reviewing groups of the 5% of films reviewed by all investigators. The Kappa level of agreement was deemed to be acceptable if above moderate (>0.60), and if less, the study would need to be redesigned [39].

## Results

**Figure 1:**
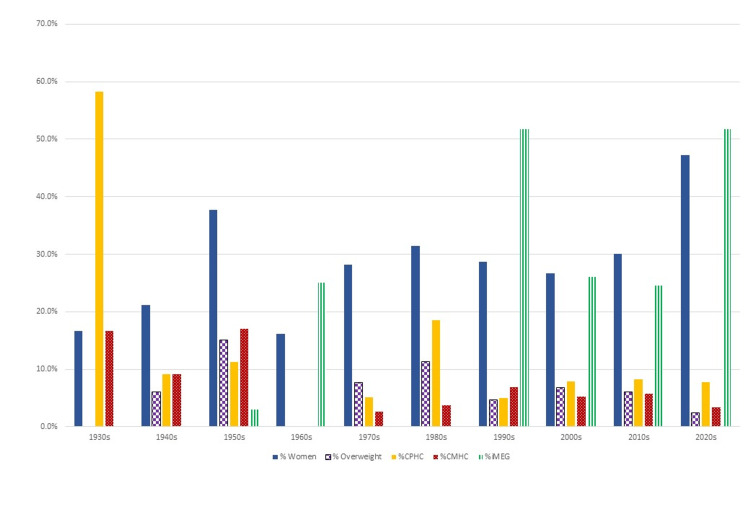
Proportion of different protected characteristics portrayed over time CPHC: characters with physical health conditions, CMHC: characters with mental health conditions, IMEG: individuals from a minority ethnic group

**Figure 2:**
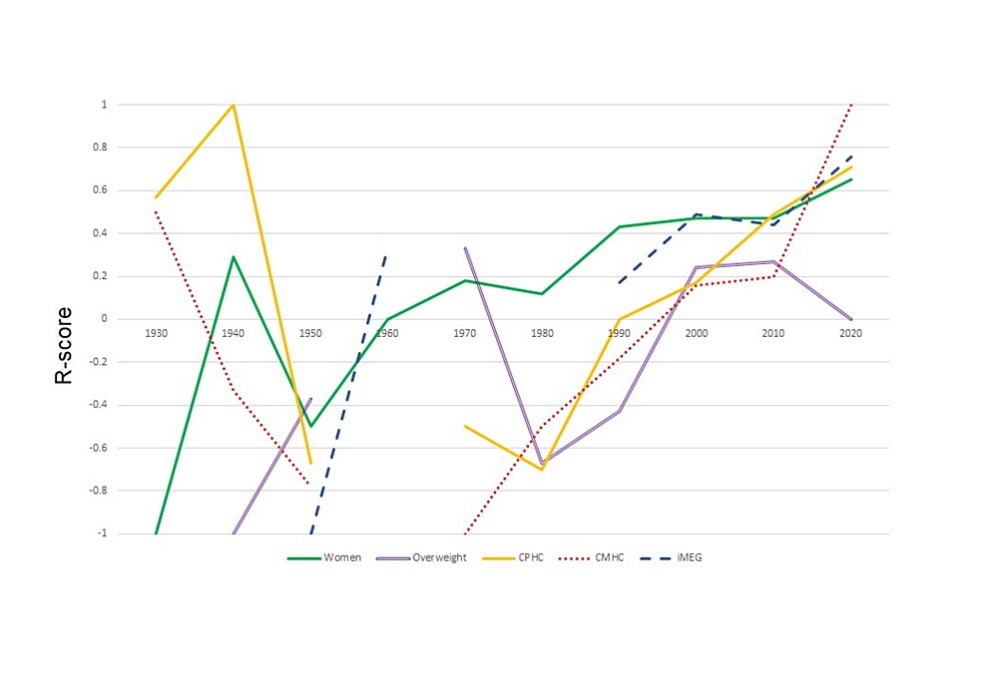
Portrayal of different protected characteristics over time CPHC: characters with physical health conditions, CMHC: characters with mental health conditions, IMEG: individuals from a minority ethnic group

In total, 116 films were reviewed with 1,275 characters included. The assessment period was from Snow White and the Seven Dwarfs in 1937 to Encanto in November 2021. Films were grouped by decade for analysis. There was good agreement (k=0.649 (95% CI. 0.647-0.652), p<0.0005) between reviewers when comparing the characters with the biggest impact on the storyline and moderate agreement when comparing characters with a smaller role (k=0.522 (95% CI. 0.520-0.523), P<0.0005) (Figures 1-2).

From the 1930s to the 2020s, the proportion of women in films increased from 16.7% to 47.3% (p=0.008) as did the proportion of women in significant roles (20.0% to 49.3%, p=0.007). After 1990, compared to pre-1990, the way in which women were represented also improved (mean R-score = -0.10 (SD:0.692) versus 0.49 (SD:0.837), p<0.001, respectively) (Figures 1-2).

The presence of overweight characters has always remained below 15.1% and since the 2000s has been on a steady decline (6.8% to 2.4%, p=0.31). During the 1980s and 1990s, there was a marked negative portrayal of overweight characters (mean R-score: -0.67 and -0.43, respectively) which changed by the 2020s to a neutral portrayal (mean R-score: -0.67 to 0.00, P=0.021) (Figures 1-2).

The proportion of characters with physical health conditions (CPHC) did not significantly change over time from the 1940s to the 2020s (9.1 to 7.7%, p=0.065). After the 1990s, the portrayal of these characters improved (mean R-score: -0.18 to 0.34, p=0.004). However, Snow White and the Seven Dwarves (1937) was unique in its greater representation of CPHC (58.3%) (Figures 1-2).

Characters with mental health conditions (CMHC) in animated films declined between the 1930s and 2020s (16.7% to 3.3%, p=0.016). There has been an improvement in the portrayal of CMHC (mean R-score = 0.5 to 1.0, p= 0.019) (Figures 1-2).

The first IMEG, the Indian Chief in Peter Pan (1953), was a negative representation, but the proportion of IMEG has increased from 3.0% in the 1950s to 51.8% by the 2020s (p<0.005). Their representation has also improved (mean R-score = -1 to 0.76, p=0.008) (Figures 1-2).

The only explicitly mentioned member of the lesbian, gay, bisexual, transgender, and queer (LGBTQ) community is Officer Spector in Onward (2020) who is gay. She was rated as a positive role model.

The most diverse film was Encanto (2021) (C-score total = 24 and FTR-score total = 24) (Table 1).

**Table 1:** Highest-scoring films depicting diversity C-score (0 the least diverse and 5 the most) determined by characters' gender, physical and mental health, ethnicity, and sexuality. FTR score based on the C-score multiplied by whether they were a positive or negative role model. FTRC-score weighted the main characters by doubling their scores.

	Total of C-score	Total of FTR-score	Total of FTRC-score	Year	Decade
Encanto	24	24	29	2021	2020
Coco	19	5	6	2017	2010
Hercules	15	-2	-3	1997	1990
The Prince of Eygpt	15	5	5	1998	1990
Peter pan	14	-9	-12	1953	1950
Mulan	14	0	2	1998	1990
Princess and the Frog	13	5	8	2009	2000
Boss baby	13	5	5	2017	2010
Bee Movie	12	0	0	2007	2000
Raya and the Last Dragon	12	4	7	2021	2020

## Discussion

This is the first study to comprehensively assess the diversity of animated film characters from the 1930s to the modern era in Disney, Pixar, and Dreamworks productions. Over time, the portrayal of women as positive role models has improved. The proportion of overweight characters has been on a steady decline over the last two decades though the way they are portrayed has improved to a neutral position. Both physical illness and mental illness are better portrayed. IMEG no longer plays only negative roles.

It is difficult to assess what direct causal effects the media has on children. However, the change in Disney Princesses from damsels in distress to independent women may help change the beliefs of what is possible for an individual [40,41]. Women have been more positively represented in animated films since the 1970s which likely reflects changing societal views. This also correlates with the greater number of women in medical schools after 1996 [41]. To further encourage women in STEM, perhaps more STEM role models are needed, like Gogo Tamago in Big Hero Six (2010) the engineering student.

Over 90% of food marketed to children uses cartoon characters, which are often encouraging the consumption of foods less healthy than alternative products aimed at adults [42]. It is interesting to note that while the number of overweight individuals in animated films has remained relatively low, the prevalence of obesity has risen [43]. In our study, earlier portrayals of overweight characters were negative, which is associated with lower levels of self-esteem [44]. Our study has shown that, over time, this trend has reversed to one where overweight characters are portrayed in a more neutral way with encouragement to be active and love your body for its shape. This is best exemplified by Po in Kung Fu Panda (2008). Obesity stigma can harm people’s health; the authors are against fat-shaming and applaud a more neutral representation of overweight characters to encourage individuals to be both proud of their bodies and strive to be the healthiest they can be [45].

CMHC appears to be in decline in the animated films we assessed, which is in stark contrast to the recent UK junior doctors report which suggested that 60% are currently suffering from depression, anxiety, stress, burnout, emotional distress, or another mental health condition [46].

Ethnic discrimination is present within the NHS and its disciplinary bodies [47]. Perhaps there is something to be learned from Disney, Pixar, and Dreamworks.

It is encouraging to see the first character from the LGBTQ community in an animated film (Onward 2020), though it must be recognized that she only had a small role. Unfortunately, in many countries, the film has been banned or her sexual orientation made ambiguous [48,49]. Some of the issues will be related to the legality of non-heterosexual behavior previously and in some countries still being illegal. Interestingly, so is murder (seen in The Lion King), attempted murder (such as with a poisonous apple), or stealing which are commonly portrayed in animated films. It should also be noted that LGBTQ individuals experience poorer health outcomes [50,51]. Further positive LGBTQ characters with larger roles should be introduced to animated films.

One of the weaknesses of our study is the subjective nature of identifying some characteristics and whether an individual character was positive, negative, or neutral. This has been mitigated by scoring characteristics only when there is no reasonable doubt and characteristics such as sexuality were explicitly stated. In this way, assumptions were not made that could incorrectly measure a greater level of diversity than present. As a result, the study maintains validity, as it is reflective of a general audience who would be less thorough in making their assessment. Of note, the level of agreement increased when only using main characters which suggests that while there may be disagreement for characters with less screen time, this may be due to limited opportunity for characters to express themselves. Furthermore, given each group was allocated an even proportion of films from each decade, the trends are likely to be a true reflection of change. Another limitation is that this study focuses on only three Western film production companies up to 2021 and does not assess other important media such as anime. It may be that diversity has continued to improve since then and in anime the number of diverse characters is greater. This would, however, need further assessment as to whether the diverse characters are also role models.

## Conclusions

In conclusion, although a slow journey, diversity in animated films is improving, and we hope this will encourage individuals, whether healthcare professionals or not, to recognize and celebrate diversity. The portrayal of women as positive role models has improved, and both physical illness and mental illness are better portrayed. IMEG no longer plays only negative roles, and there is now an explicitly stated homosexual character portrayed. We hope that the direction of travel toward a more diverse cast of positively portrayed characters, like in Encanto, will continue. In the global community, we need to challenge inequalities and encourage diversity. We hope that the trends identified in animated films can also be mirrored in the healthcare community, to develop and better serve the diverse population.
